# Chromosome-level genome assembly of an auxotrophic strain of the pathogenic yeast *Candida parapsilosis*

**DOI:** 10.1128/mra.00347-24

**Published:** 2024-07-31

**Authors:** Sofia Mutalová, Viktória Hodorová, Filip Brázdovič, Andrea Cillingová, Ľubomír Tomáška, Broňa Brejová, Jozef Nosek

**Affiliations:** 1 Department of Biochemistry, Faculty of Natural Sciences, Comenius University Bratislava, Bratislava, Slovak Republic; 2 Laboratory of Regulation of Gene Expression, Institute of Microbiology of the Czech Academy of Sciences, Prague, Czech Republic; 3 Department of Genetics, Faculty of Natural Sciences, Comenius University Bratislava, Bratislava, Slovak Republic; 4 Department of Computer Science, Faculty of Mathematics, Physics and Informatics, Comenius University Bratislava, Bratislava, Slovak Republic; University of California Riverside, Riverside, California, USA

**Keywords:** yeasts, *Candida*, genome analysis

## Abstract

We report the genome sequence of the pathogenic yeast *Candida parapsilosis* strain SR23 (CBS 7157) used in a number of experimental studies. The nuclear genome assembly consists of eight chromosome-sized contigs with a total size of 13.04 Mbp (N50 2.09 Mbp) and a G+C content of 38.7%.

## ANNOUNCEMENT

*Candida parapsilosis* SR23 is an adenine and lysine auxotroph isolated about 40 years ago from contaminated culture of *Saccharomyces cerevisiae* as a yeast with peculiar physiological features and carrying a linear mitochondrial DNA (mtDNA) becoming one of the pillars for a systematic analysis of mtDNA architecture in yeasts (1, 2). Later studies showed that the linear mtDNA and its telomeric structures represent a typical feature of the “*psilosis*” group species (3–8). Importantly, SR23 became a model for investigations of telomere protection and telomerase-independent replication (9–16), and it was also instrumental in the development of tools for genetic manipulation of *C. parapsilosis* (17–19). To facilitate further studies, we determined the genome sequence of this strain originating from the laboratory of P. P. Slonimski (Gif-Sur-Yvette, France) and provided to us by L. Kováč in 1987.

High-molecular-weight DNA was isolated as described previously (20) from an overnight culture grown in yeast extract–peptone–dextrose (YPD) medium (Table 1) at 28°C and, without shearing or size selection, used for the preparation of a ligation library and sequenced in two runs on a MinION device with an R9.4.1 flow cell [Oxford Nanopore Technologies (ONT)]. The resulting data (Table 1) were basecalled using Guppy v.6.4.2 (ONT) in the HAC mode and quality-checked by NanoPlot v.1.33.1 (21). DNA was also sequenced on NovaSeq 6000 (Table 1), and the quality of the reads was checked by FastQC v.0.12.1 (22).

**TABLE 1:** Sequencing data used in the assembly and annotation of the *C. parapsilosis* SR23 genome

Sample	Sequencing platform^a^	Library kit	Total amount (Gbp)	Number of reads	ENA accession number	Cultivation medium
DNA	MinION Mk1B	SQK-LSK109	4.96	659,024 (N50 = 13,492 nt)	ERR12736264, ERR12736265	YPD [1% (wt/vol) yeast extract, 2% (wt/vol) peptone, 2% (wt/vol) glucose]
DNA	NovaSeq 6000	TruSeq DNA PCR-free (350 bp), paired ends (2 × 151 nt)	5.62	37,243,512	ERR12736259	YPD [1% (wt/vol) yeast extract, 2% (wt/vol) peptone, 2% (wt/vol) glucose]
RNA	NovaSeq 6000	TruSeq Stranded mRNA LT Sample Prep, paired ends (2 × 151 nt)	4.32	28,597,422	ERR12736360	Synthetic with 4-hydroxybenzoate [0.17% (wt/vol) yeast nitrogen base without amino acids and ammonium sulfate (Difco), 0.5% (wt/vol) ammonium sulfate, 10 mM 4-hydroxybenzoate]
RNA	NovaSeq 6000	TruSeq Stranded mRNA LT Sample Prep, paired ends (2 × 151 nt)	6.54	43,330,142	ERR12736361	Synthetic with galactose [0.17% (wt/vol) yeast nitrogen base without amino acids and ammonium sulfate (Difco), 0.5% (wt/vol) ammonium sulfate, 2% (wt/vol) galactose]
RNA	NovaSeq 6000	TruSeq Stranded mRNA LT Sample Prep, paired ends (2 × 151 nt)	6.27	41,547,686	ERR12736362	Synthetic with hydroquinone [0.17% (wt/vol) yeast nitrogen base without amino acids and ammonium sulfate (Difco), 0.5% (wt/vol) ammonium sulfate, 10 mM hydroquinone]

^a^ Sequencing on Illumina NovaSeq 6000 platform was performed by Macrogen Europe (Amsterdam, the Netherlands).

**Fig 1:**
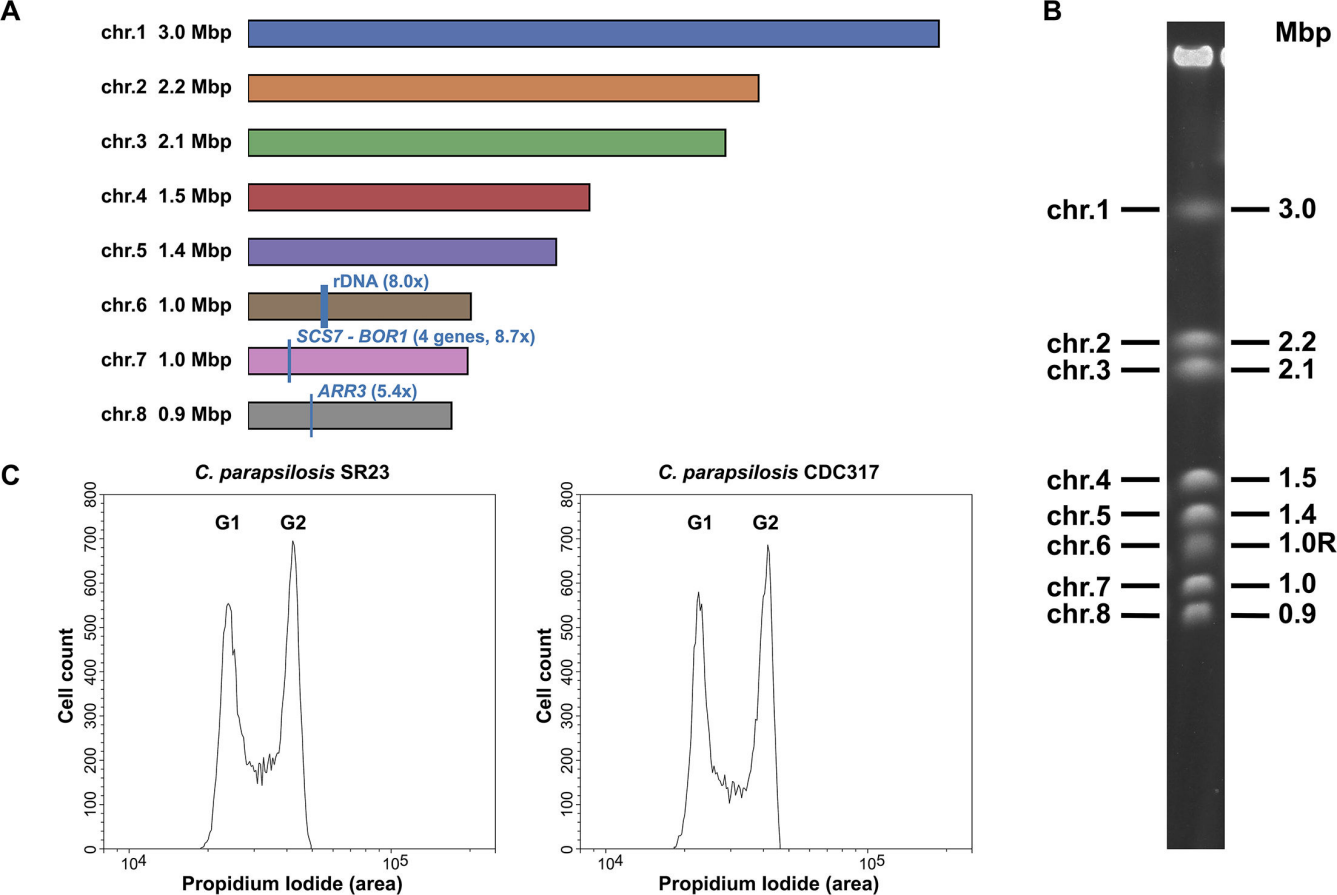
(**A**) Nuclear chromosomes of *C. parapsilosis* SR23 visualized using Matplotlib library in Python (28). Genome regions with Illumina coverage higher than the overall median are shown in blue; these include rDNA (8.0×) and two segments with four genes (*SCS7*/CPARSR23_p51490, CPARSR23_p51500, CPARSR23_p51510, and *BOR1*/CPARSR23_p51520; 8.7×) and the gene *ARR3*/CPARSR23_p56030 (5.4×) on chromosomes 6, 7, and 8, respectively. (**B**) Electrophoretic karyotype of *C. parapsilosis* SR23. Chromosomal DNA was separated by pulsed-field gel electrophoresis in a 0.8% (wt/vol) agarose gel in 0.5× Tris-borate-Ethylenediaminetetraacetic acid (TBE) buffer (45 mM Tris-borate, 1 mM EDTA) using a Pulsaphor system (LKB) in contour-clamped homogeneous electric field configuration with pulse switching from 60 to 600 s for 72 h at 100 V and 9°C throughout (5). Note that chromosome 6 (labeled as 0.98R) contains multiple rDNA repeats and, therefore, appears longer than in the assembly. (**C**) Flow cytometry confirms the diploid nature of *C. parapsilosis* SR23 cells. DNA content was analyzed on a CytoFLEX S flow cytometer (Beckman Coulter) essentially as described in references (29, 30). Histograms show DNA content [propidium iodide (area)] vs cell counts. The peaks represent the cells in G1 and G2 phases of the cell cycle at the time of fixation. Diploid cells of the reference strain *C. parapsilosis* CDC317 (31) were used as a control.

Flye v.2.9.1-b1780 (23) was run on 40% of nanopore reads longer than 5 kbp. Nanopore reads were aligned to the assembly by minimap2 v.2.24-r1122 (24), and the assembly was manually adjusted based on the inspection of read alignments. Namely, low-quality sequences were truncated from two contigs, and one contig was split at a telomere. Two chromosomes were subsequently created by joining parts of two or three contigs, respectively. Mitochondrial DNA was replaced by the published version NC_005253.2 (4). Subsequently, the whole assembly was polished by three iterations of Pilon v.1.21 (25) using Illumina reads aligned by bwa mem v.0.7.17-r1188 (26). Copies of the rDNA repeat were replaced by a separately polished full-length copy. The nuclear genome assembly contains eight contigs with telomeres (5′-CCGGATGTTGATTATACTGAGGT-3′)_*n*_ (27) on both ends corresponding to the electrophoretic karyotype and, thus, likely represent full-length chromosomes. Moreover, flow cytometry indicates that SR23 cells are diploid (Fig. 1).

For RNA-seq, SR23 cultures grew in three synthetic media differing by the carbon source (Table 1) at 28°C till optical density (OD)_600_ ~1. Total RNA was extracted using hot phenol (32), purified by an RNeasy Mini kit (Qiagen), and sequenced on NovaSeq 6000 (Table 1). The reads were trimmed by Trimmomatic v.0.36 (33) and assembled to transcripts by Trinity v.2.15.1 (34). These were mapped to the genome by blat v.36×7 (35) and used as evidence to predict protein-coding genes by Augustus v.3.2.3 (36) with species-specific parameters previously trained on *C. parapsilosis* CLIB214 (37). We manually adjusted 342 genes based on a comparison with RNA-seq data and genes from other strains. In total, 5,879 protein-coding genes were annotated in the nuclear genome. Full details of bioinformatics analyses can be found on Zenodo (38).

## Data Availability

The genome assembly has been deposited in the European Nucleotide Archive (ENA)/GenBank databases with accession number GCA_963989715. The genome sequence version described in this paper is the first version, GCA_963989715.1. The ENA accession numbers of Illumina and nanopore reads are shown in Table 1. The mitochondrial genome sequence was published previously (4) and is also available in GenBank and ENA databases under accession numbers NC_005253.2 and X74411.6, respectively. The results are also presented in a genome browser at http://genome.compbio.fmph.uniba.sk/.
